# The financial burden of alopecia: a survey study

**DOI:** 10.1097/JW9.0000000000000118

**Published:** 2023-11-01

**Authors:** Jenne P. Ingrassia, Michael G. Buontempo, Lina Alhanshali, Christine C. Akoh, Sharon Glick, Jerry Shapiro, Kristen Lo Sicco

**Affiliations:** a Ronald O. Perelman Department of Dermatology, New York University Grossman School of Medicine, New York City, New York; b Department of Undergraduate Medical Education, School of Medicine, New York Medical College, Valhalla, New York; c Department of Dermatology, Hackensack Meridian School of Medicine, Nutley, New Jersey; d Department of Undergraduate Medical Education, SUNY Downstate School of Medicine, Brooklyn, New York

**Keywords:** alopecia, camouflaging agents, cranial prostheses, dermatologist, financial burden, hair loss, health insurance coverage, medical expenses, over-the-counter products, psychosocial burden, treatment options

**What is known about this subject in regard to women and their families?**

- Alopecia, a chronic hair loss condition, has significant psychological, financial, self-identity, and quality-of-life impacts, frequently incurring considerable financial costs for individuals and their families.
- Women and their families often resort to alternative therapies, such as over-the-counter products, camouflaging agents, or cranial prostheses.

**What is new from this article as messages for women and their families?**

- The study reveals that individuals with alopecia, predominately women, face significant out-of-pocket expenses for treatments and measures to conceal their condition.
- The lack of insurance coverage for alopecia-related costs adds to the financial stress, with 45% of individuals with nonscarring alopecia reporting mild to extreme stress due to this lack of coverage.
- The study underlines the lack of health insurance coverage for most alopecia treatments, resulting in substantial out-of-pocket expenditures for patients.

## Dear Editors,

Alopecia, an often chronic hair loss condition, impacts individuals’ self-identity, psychological well-being, and quality of life, and may incur considerable financial costs.^1–4^ Patients frequently turn to alternative therapies, such as over-the-counter (OTC) products, camouflaging agents, or cranial prostheses.^1–3^ This study explores the financial burden experienced by alopecia patients.

An Institutional Review Board–approved cross-sectional survey was carried out from March to May 2023, targeting patients visiting a hair loss clinic in New York City. The survey assessed patient demographics, types of hair loss, a range of OTC and adjunctive treatments, as well as the associated medical expenses. The Python programming language facilitated descriptive statistical analysis and pairwise *χ*^2^ tests. Adjusted residuals, odds ratios, and confidence intervals were employed to investigate potential cost determinants, considering a *P* < .05 as statistically significant.

A total of 100 surveys were administered with a 100% response rate. Baseline characteristics were: 64% female, 76% white, 60% aged 25–44, and 98% medically insured. The majority of participants (70%) had nonscarring alopecia. Ten participants (10%) had scarring alopecia, and 20 (20%) had an unclear diagnosis or did not answer. All patients had chronic alopecia (>6 months).

Within the past year, 46 (66%) participants with nonscarring alopecia purchased at least 1 OTC hair growth product, with 74% (34 of 46) reporting no improvement or uncertain effectiveness. Comparatively, 8 (80%) of participants with scarring alopecia reported that they purchased at least 1 OTC hair growth product, with 88% (7 of 8) reporting no improvement or uncertain effectiveness. In the past year, 17 (24%) participants with nonscarring alopecia reported spending over $500 dollars on OTC hair growth agents. In contrast, 5 (50%) of participants with scarring alopecia spent over $500 dollars on OTC hair growth products. Thirty-one (44%) participants with nonscarring alopecia purchased camouflaging agents, with 5 (16%) spending over $500 on these items over the past year (Table 1). Insurance did not cover the costs for nearly all of those who purchased camouflaging agents (22 of 31), with 45% (10 of 22) reporting mild to extreme stress due to lack of coverage. For participants with scarring alopecia, 7 (70%) used camouflaging agents, and 5 (50%) spent over $500 on these agents (Table 2). Insurance did not cover the costs for nearly all of those who purchased camouflaging agents (6 of 7), with 83% (5 of 6) reporting mild to extreme stress due to lack of coverage.

**Table 1:** Reported spending on nondermatologist providers for hair loss, over-the-counter products, and camouflaging agents within the last year for patients with nonscarring alopecia

Category of spending	*n* (%)
Nondermatologist providers	
Under $50	35 (50)
$51 to $100	3 (4)
$101 to $250	7 (10)
$251 to $500	5 (7)
Over $500	18 (26)
Did not answer	1 (1)
Over-the-counter products	
Under $50	7 (10)
$51 to $100	6 (9)
$101 to $250	15 (21)
$251 to $500	9 (13)
Over $500	24 (34)
Did not answer	0 (0)
Did not purchase	8 (11)
Camouflaging agents	
Under $50	14 (20)
$51 to $100	4 (6)
$101 to $250	5 (7)
$251 to $500	0 (0)
Over $500	6 (9)
Did not answer	0 (0)
Did not purchase	39(56)

**Table 2:** Reported spending on nondermatologist providers for hair loss, over-the-counter products, and camouflaging agents within the last year for patients with scarring alopecia

Category of spending	*n* (%)
Nondermatologist providers	
Under $50	6 (60)
$51 to $100	0 (0)
$101 to $250	2 (20)
$251 to $500	0 (0)
Over $500	2 (20)
Did not answer	0 (0)
Over-the-counter products	
Under $50	0 (0)
$51 to $100	2 (20)
$101 to $250	1 (10)
$251 to $500	0 (0)
Over $500	5 (50)
Did not answer	2 (20)
Did not purchase	0 (0)
Camouflaging agents	
Under $50	1 (10)
$51 to $100	0 (0)
$101 to $250	1 (10)
$251 to $500	1 (10)
Over $500	5 (50)
Did not answer	1(10)
Did not purchase	1(10)

Participants with scarring and nonscarring alopecia who answered that they felt concerned about their hair every day were more likely to spend over $500 on nondermatologist providers before consulting an alopecia specialist dermatologist (OR = 3.90; 95% CI [1.51–10.10]). Notably, 8% reported that hair product expenditures hindered them from purchasing basic necessities or desired items. Additionally, a greater proportion of patients with scarring alopecia spent over $500 dollars on OTC and camouflaging agents than patients with nonscarring alopecia.

The financial burden of alopecia is underexplored in the literature, with most studies focusing on alopecia areata.^2,3,5^ We found that individuals diagnosed with alopecia experience additional financial costs for treating or concealing their condition. Higher out-of-pocket expenses arose from initially seeking nondermatologist providers, purchasing OTC products, and utilizing camouflaging agents, such as cranial prostheses. Many participants used oral and topical OTC products with limited efficacy, and the financial burden associated with hair growth products and camouflaging agents notably affected their ability to purchase basic items of daily living. While our sample size of participants with scarring alopecia was limited, the findings suggest that illness type, such as scarring versus nonscarring alopecia, may impact spending patterns on OTC and camouflaging agents.

These findings are valuable for dermatologists, as they highlight the financial burden experienced by alopecia patients. Similar to data from alopecia areata patients, we observed high costs associated with wigs, headwear, and other camouflaging agents.^5^ To alleviate the psychosocial and economic burden associated with alopecia management, wider health insurance coverage for camouflaging agents is necessary.^3,5^ Patients should discuss financial barriers with their dermatologist during initial appointments to guide the selection of affordable treatments.

Study limitations include selection bias due to patient demographics likely representing a higher socioeconomic status due to sampling patients at a tertiary care center, which often sees moderate to severe alopecia cases, and undercoverage bias due to satisfied OTC users being less likely to seek care. The limited diversity of our participant population with the majority being insured, and the limited sample size of patients with scarring alopecia are additional key limitations. Larger scale studies are necessary to further quantify the difference in spending for scarring and nonscarring alopecia.

## Conflicts of interest

J.S. is a consultant for Aclaris Therapeutics, Incyte, and Replicel Life Sciences. J.S. and K.L.S. have been investigators for Regen Lab and are investigators for Pfizer. K.L.S. is a consultant for Pfizer and Aquis. The other author has no conflicts of interest to disclose.

## Funding

None.

## Study approval

This study was approved by the NYU Langone Institutional Review Board on March 14, 2023; approval #: s23-00099.

## Author contributions

JPI and MGB: Study design, data collection, and manuscript writing. LA: Study design, data collection, and manuscript editing. CCA: Manuscript writing. SG: Manuscript editing. JS: Project oversight and manuscript editing. KLS: Study design, project oversight, and manuscript editing.
